# Elevated HbA1c remains a predominant finding in severe COVID-19 and may be associated with increased mortality in patients requiring mechanical ventilation

**DOI:** 10.1186/s13054-021-03730-2

**Published:** 2021-08-19

**Authors:** Sebastian J. Klein, Timo Mayerhöfer, Dietmar Fries, Christian Preuß Hernández, Michael Joannidis, Collaborators, Romuald Bellmann, Andreas Peer, Julia Haßlacher, Adelheid Ditlbacher, Georg F. Lehner, Fabian Perschinka, Walter Hasibeder, Christoph Krismer, Agnes Pechlaner, Stephan Eschertzhuber, Stefanie Zagitzer-Hofer, Eva Foidl, Isabella Weilguni, Stefanie Haslauer-Mariacher, Armin Kalenka, Alexandra Ribitsch, Andreas Mayr, Eugen Ladner, Bernhard Mayr-Hueber, Birgit Stögermüller, Lukas Kirchmair, Bruno Reitter, Miriam Potocnik, Simon Mathis, Anna Fiala, Jürgen Brunner, Claudius Thomé

**Affiliations:** 1 Division of Intensive Care and Emergency Medicine, Department of Internal Medicine, Medical University Innsbruck, Anichstrasse 35, 6020 Innsbruck, Austria; 2 Department of Anesthesia and Critical Care Medicine, Medical University Innsbruck, Innsbruck, Austria; 3 Department of Neurosurgery, Medical University Innsbruck, Innsbruck, Austria

## Dear Editor,

We have previously reported unusually high rates of elevated glycated hemoglobin (HbA1c) levels in patients with COVID-19 admitted to an ICU between March 11 and April 29, 2020 [1]. Since then, our Tyrolean multicenter COVID-19 Intensive Care Unit Registry (Tyrol-CoV-ICU-Reg) [2] has considerably increased, surpassing 500 patients. To re-evaluate our previously reported findings, we included 306 additional patients in this analysis, who were admitted between April 30, 2020 and May 31, 2021, for whom an admission HbA1c was available. Details of our registry have been reported before [2].

Admission HbA1c was now available in 350 patients. We were able to confirm our finding that HbA1c was elevated (i.e., HbA1c ≥ 5.7%) in most patients (85.1%). However, only 31.7% had a history of diabetes mellitus (DM) or prediabetes (Table 1). Median HbA1c at admission was significantly higher in patients with HbA1c ≥ 6.5% and a history of DM than without history of DM (*Mann–Whitney U* p < 0.001). Furthermore, a weak correlation for HbA1c and BMI could be established (*Pearson* R = 0.27, p < 0.001).

**Table 1:** General characteristics of included patients stratified by history of diabetes mellitus/prediabetes and HbA1c

	Overall	No history of diabetes mellitus/prediabetes			History of diabetes mellitus/prediabetes		
		HbA1c < 5.7%	HbA1c 5.7 < 6.5%	HbA1c ≥ 6.5%	HbA1c < 5.7%	HbA1c 5.7 < 6.5%	HbA1c ≥ 6.5%
n	350	49	138	52	3	18	90
Age [years] (median [IQR])	68.00 [58.00, 76.00]	67.00 [54.00, 77.00]	70.00 [61.00, 77.00]	65.00 [55.50, 75.25]	63.00 [56.50, 68.00]	72.00 [62.50, 74.75]	68.00 [58.25, 76.75]
Male—no. (%)	250 (71.4)	32 (65.3)	102 (73.9)	40 (76.9)	3 (100.0)	11 (61.1)	62 (68.9)
BMI [kg/m2] (median [IQR])	27.76 [25.14, 31.22]	25.12 [22.10, 27.53]	27.47 [24.97, 30.66]	29.45 [26.41, 31.51]	25.13 [24.18, 29.52]	28.73 [26.93, 31.71]	29.39 [26.51, 32.92]
HbA1c [%] (median [IQR])	6.30 [5.90, 6.80]	5.40 [5.10, 5.50]	6.10 [5.90, 6.20]	6.70 [6.60, 7.12]	5.50 [5.50, 5.55]	6.10 [5.93, 6.30]	7.60 [6.80, 8.88]
IMV—no. (%)	194 (55.4)	28 (57.1)	80 (58.0)	31 (59.6)	1 (33.3)	9 (50.0)	45 (50.0)
AKI—no. (%)							
no AKI	233 (67.0)	33 (68.8)	94 (68.6)	31 (59.6)	2 (66.7)	12 (66.7)	61 (67.8)
KDIGO I	38 (10.9)	4 (8.3)	16 (11.7)	7 (13.5)	0 (0.0)	2 (11.1)	9 (10.0)
KDIGO II	18 (5.2)	2 (4.2)	7 (5.1)	2 (3.8)	0 (0.0)	1 (5.6)	6 (6.7)
KDIGO III	59 (17.0)	9 (18.8)	20 (14.6)	12 (23.1)	1 (33.3)	3 (16.7)	14 (15.6)
RRT—no. (%)	55 (15.7)	9 (18.4)	18 (13.0)	10 (19.2)	1 (33.3)	5 (27.8)	12 (13.3)
vv-ECMO	18 (5.1)	4 (8.2)	5 (3.6)	6 (11.5)	0 (0.0)	0 (0.0)	3 (3.3)
IMV [days] (median [IQR])	14.0 [8.0, 24.3]	10.5 [5.5, 20.8]	13.0 [8.0, 25.3]	15.00 [12.5, 26.50]	7.0 [7.0, 7.0]	16.0 [5.0, 29.0]	13.0 [8.0, 24.0]
RRT [days] (median [IQR])	11.0 [3.0, 25.5]	12.0 [6.0, 13.0]	16.5 [3.5, 27.5]	13.50 [9.5, 25.8]	7.0 [7.0, 7.0]	1.0 [1.0, 4.0]	4.0 [1.8, 14.5]
ECMO [days] (median [IQR])	23.5 [12.5, 28.8]	15.0 [14.0, 16.5]	26.0 [14.0, 26.0]	28.50 [16.0, 38.0]	NA [NA, NA]	NA [NA, NA]	27.0 [19.5, 28.5]
SAPS III score (median [IQR])	55.0 [48.0, 63.0]	59.0 [49.5, 69.8]	54.5 [49.0, 63.3]	52.50 [47.0, 62.5]	53.0 [49.5, 61.5]	55.5 [50.8, 59.3]	55.0 [48.0, 62.0]
Hospital LOS (median [IQR])	23.0 [14.0, 39.5]	26.5 [14.0, 40.0]	22.0 [15.0, 35.0]	24.00 [12.0, 45.5]	44.0 [33.5, 61.5]	30.5 [14.8, 45.5]	21.0 [13.0, 36.5]
ICU LOS (median [IQR])	11.0 [5.0, 23.0]	10.0 [4.0, 23.0]	13.0 [7.0, 23.0]	16.50 [5.0, 29.0]	5.0 [3.5, 10.5]	9.5 [6.0, 30.5]	10.0 [5.0, 21.0]
Known comorbidity*—no. (%)							
Cardiovascular	142 (40.6)	21 (42.9)	56 (40.6)	18 (34.6)	2 (66.7)	12 (66.7)	33 (36.7)
Arterial hypertension	221 (63.1)	26 (53.1)	82 (59.4)	25 (48.1)	3 (100.0)	15 (83.3)	70 (77.8)
Renal	78 (22.3)	12 (24.5)	24 (17.4)	10 (19.2)	2 (66.7)	8 (44.4)	22 (24.4)
Liver	28 (8.0)	3 (6.1)	12 (8.7)	6 (11.5)	0 (0.0)	1 (5.6)	6 (6.7)
Metastatic disease	2 (0.6)	0 (0.0)	0 (0.0)	1 (1.9)	0 (0.0)	0 (0.0)	1 (1.1)
Hematological malignancy	18 (5.1)	3 (6.1)	7 (5.1)	2 (3.8)	0 (0.0)	0 (0.0)	6 (6.7)
Non-hematological malignancy	28 (8.0)	3 (6.1)	15 (10.9)	3 (5.8)	0 (0.0)	0 (0.0)	7 (7.9)
COPD	17 (4.9)	4 (8.2)	8 (5.8)	0 (0.0)	0 (0.0)	1 (5.6)	4 (4.4)
Asthma	50 (14.3)	4 (8.2)	18 (13.0)	8 (15.4)	1 (33.3)	2 (11.1)	17 (18.9)
Respiratory disease—others	27 (7.7)	2 (4.1)	12 (8.7)	3 (5.8)	0 (0.0)	2 (11.1)	8 (8.9)
Diabetes mellitus—no. (%)							
No pre-known dysglycemia	239 (68.3)	49 (100.0)	138 (100.0)	52 (100.0)	0 (0.0)	0 (0.0)	0 (0.0)
Prediabetes	12 (3.4)	0 (0.0)	0 (0.0)	0 (0.0)	0 (0.0)	4 (22.2)	8 (8.9)
DM Type I	4 (1.1)	0 (0.0)	0 (0.0)	0 (0.0)	0 (0.0)	2 (11.1)	2 (2.2)
DM Type II	94 (26.9)	0 (0.0)	0 (0.0)	0 (0.0)	3 (100.0)	11 (61.1)	80 (88.9)
DM (other Type, e.g., MODY)	1 (0.3)	0 (0.0)	0 (0.0)	0 (0.0)	0 (0.0)	1 (5.6)	0 (0.0)
ICU mortality—no. (%)	90 (25.7)	13 (26.5)	35 (25.4)	14 (26.9)	0 (0.0)	3 (16.7)	25 (27.8)
Hospital mortality—no. (%)	100 (28.6)	15 (30.6)	37 (26.8)	15 (28.8)	0 (0.0)	3 (16.7)	30 (33.3)

Of 350 included patients, 44 have been previously reported [1]

*IQR* interquartile range, *BMI* body mass index, *HbA1c* glycated hemoglobin, *IMV* invasive mechanical ventilation, *AKI* acute kidney injury, *KDIGO* kidney disease: improving global outcomes, *RRT* renal replacement therapy, *vv-ECMO* veno-venous extracorporeal membrane oxygenation, *SAPS* simplified acute physiology score, *LOS* length of stay, *ICU* intensive care unit, *COPD* chronic obstructive pulmonary disease, *DM* Diabetes mellitus

*As mentioned in previous medical documents

There was a trend toward longer duration of invasive mechanical ventilation (IMV) in patients with higher HbA1c, albeit non-significant. In patients with history of DM, a tendency toward increased mortality associated with elevated HbA1c was observed, but the groups differed considerably in size (Table 1). When comparing patients with an HbA1c ≥ 6.5% requiring IMV to the rest of our cohort, ICU (40.8% vs. 21.5%; *Χ*^*2*^ p = 0.001) and hospital mortality (42.1% vs. 24.8%; *Χ*^*2*^ p = 0.005) were significantly increased.

In a multivariate logistic regression model including HbA1c, history of DM and IMV, odds ratios (ORs) for hospital death were higher for patients with elevated HbA1c ≥ 6.5%. However, this effect seems to be mainly driven by IMV. A significant association with hospital mortality was shown for treatment with IMV, age and SAPS III score (Table 2).

**Table 2:** Logistic regression analysis for prediction of hospital mortality

	Univariate analysis		Multivariate analysis	
	OR (95% CI)	p	OR (95% CI)	p
Age	1.08 (1.05–1.11)	< 0.001	1.07 (1.04–1.11)	< 0.001
Sex (male)	1.29 (0.77–2.21)	0.350		
BMI	1.00 (0.97–1.05)	0.737		
Number of comorbidities	1.29 (1.13–1.49)	< 0.001	1.24 (1.04–1.48)	0.018
SAPS III score	1.08 (1.05–1.11)	< 0.001	1.05 (1.02–1.08)	0.001
IMV	3.08 (1.87–5.21)	< 0.001		
*Ref: HbA1c < 6.5 and no history of DM*				
HbA1c < 6.5 and history of DM	0.43 (0.20–1.35)	0.194		
HbA1c ≥ 6.5 and no history of DM	1.05 (0.52–2.05)	0.883		
HbA1c ≥ 6.5 and history of DM	1.30 (0.75–2.23)	0.346		
*Ref: HbA1c < 6.5 and no history of DM and no IMV*				
HbA1c < 6.5 and history of DM, no IMV			0.30 (0.01–2.16)	0.304
HbA1c ≥ 6.5 and no history of DM, no IMV			0.31 (0.02–1.99)	0.298
HbA1c ≥ 6.5 and history of DM, no IMV			2.18 (0.71–6.71)	0.168
HbA1c < 6.5 and no history of DM, IMV			3.94 (1.72–9.66)	0.002
HbA1c < 6.5 and history of DM, IMV			0.78 (0.04–6.16)	0.832
HbA1c ≥ 6.5 and no history of DM, IMV			6.36 (2.02–20.85)	0.002
HbA1c ≥ 6.5 and history of DM, IMV			3.66 (1.38–10.17)	0.011

HbA1c and variables with a p value < 0.05 in univariate analysis were included in multivariate analysis. Of 350 included patients, 44 have been previously reported [1]

*BMI* body mass index, *SAPS* simplified acute physiology score, *IMV* invasive mechanical ventilation, *HbA1c* glycated hemoglobin, *DM* diabetes mellitus, *OR* odds ratio, *CI* confidence interval, *Ref* reference category

In this follow-up analysis, including 306 additional cases, we were able to confirm our previous findings [1] of an extremely high incidence of elevated HbA1c in critically ill COVID-19 patients. While other pre-admission comorbidities (e.g., arterial hypertension) were more common than DM, chronic dysglycemia as defined by an HbA1c ≥ 5.7% was the predominant factor in our cohort of critically ill COVID-19 patients. We still did not find a strong association between elevated HbA1c and ICU/hospital mortality, but in the subgroup of patients requiring mechanical ventilation, mortality was significantly increased in patients with HbA1c ≥ 6.5%. This is in line with other cohorts of COVID-19 patients, showing that admission blood glucose may be a more relevant predictor for mortality than HbA1c [3]. Admission blood glucose may be interpreted as a biomarker of systemic inflammation on admission, whereas HbA1c represents a marker of glucose control of the past three months [4, 5]. Unfortunately, blood glucose measurements during ICU admission were not recorded in our registry. Furthermore, follow-up of reported patients would be necessary to confirm diagnosis of previously unrecognized diabetes mellitus by admission HbA1c. We also have to acknowledge a potential selection bias for patients in whom HbA1c was measured. BMI has recently been reported as an important risk factor for severe COVID-19 [3]. Though we could establish a weak correlation between BMI and HbA1c, we want to emphasize that prediabetes/and diabetes may exist independently and thus remain an independent risk factor for ICU admission in COVID-19. A possible explanation may be a pathological inflammatory response in diabetic patients [6]. When exposed to an additional inflammatory stimulus, such as mechanical ventilation on top of COVID-19, outcome may be impaired. This finding warrants further research in terms of risk stratification at ICU admission.

## Data Availability

No data are publicly available at this time.
